# Machine learning‐based integration of radiomics and dosiomics for early prediction of radiation‐induced temporal lobe injury in nasopharyngeal carcinoma: A multicenter study

**DOI:** 10.1002/acm2.70420

**Published:** 2025-12-28

**Authors:** Xiaoming Sun, Huijun Zhu, Jian Qiao, Yingying Liang, Zhipeng Zhu, Sixue Dong, Jiazhou Wang

**Affiliations:** ^1^ Department of Radiation Oncology Fudan University Shanghai Cancer Center Shanghai China; ^2^ Department of Oncology, Shanghai Medical College Fudan University Shanghai China; ^3^ Shanghai Clinical Research Center for Radiation Oncology Shanghai China; ^4^ Department of Radiation Oncology The Second Affiliated Hospital of Guangxi Medical University Nanning Guangxi China

**Keywords:** dosiomics, machine learning, predictive modeling, radiation‐induced temporal lobe injury, radiomics, support vector machine

## Abstract

**Purpose:**

This study developed and validated a multimodal machine learning model integrating clinical, radiomics, and dosiomics characteristics to predict radiation‐induced temporal lobe injury (RTLI) in nasopharyngeal carcinoma patients.

**Methods:**

This study utilized dual‐center retrospective cohorts comprising 213 patients. From pre‐treatment 3D computed tomography (CT) scans and radiation dose maps, we extracted 43 radiomics features and 43 dosiomics features. These quantitative imaging features underwent a rigorous three‐stage selection pipeline—including auto‐correlation analysis, significance testing (*t*‐tests), and least absolute shrinkage and selection operator‐regularized logistic regression (LASSO‐LR)—yielding 24 optimal image‐derived predictors (13 radiomics and 11 dosiomics features). In parallel, eight clinical features were preserved in all final models due to their established clinical relevance and prior biological significance. We systematically evaluated twelve predictive configurations by integrating three machine learning algorithms—Support Vector Machine (SVM), Random Forest (RF), and Logistic Regression (LR)—with four feature modalities: clinical‐only, radiomics‐only, dosiomics‐only, and an integrated feature set.

**Results:**

The SVM‐based integrated model demonstrated superior predictive performance, achieving an AUC of 0.819 during internal validation while maintaining robust generalizability in external testing (AUC = 0.714). The model exhibited excellent specificity (0.844) and remarkable cross‐cohort stability, as evidenced by low performance variability (standard deviation: ±0.077 in validation, ±0.056 in testing). These findings highlight the synergistic value of integrating clinically established parameters with quantitative characterization of anatomical heterogeneity (radiomics) and radiation dose distribution patterns (dosiomics) for accurate prediction of radiation‐induced temporal lobe injury.

**Conclusion:**

This clinically applicable predictive framework enables early identification of high‐risk RTLI patients, facilitating timely interventions to mitigate neurotoxicity. The model's demonstrated multicenter validity, high specificity, and balanced performance across evaluation metrics address critical limitations of current prediction approaches, representing a significant advance in personalized radiation oncology for nasopharyngeal carcinoma patients.

## 1 INTRODUCTION

Radiation‐induced temporal lobe injury (RTLI) has emerged as a critical dose‐limiting toxicity in contemporary radiotherapy for head and neck cancers.^1^ The incidence and severity of RTLI have shown a concerning upward trend parallel to technological advancements in radiation delivery systems.^2^ Moreover, epidemiological data reveal a striking technology‐dependent increase in RTLI incidence: progressing from <5% with conventional radiotherapy to 8.2%–15.7% with intensity‐modulated radiation therapy (IMRT) and peaking at 18.3% in proton therapy cohorts.^3^ RTLI induces multifaceted neurotoxicity, causing cognitive decline, memory impairment, and potentially refractory epilepsy,^2^, ^4^, ^5^, ^6^ which severely impairs daily functioning and social integration. Therefore, there is a critical need for early risk identification and prediction of RTLI to facilitate the timely implementation of neuroprotective strategies and to promptly adjust individualized treatment plans—an essential research priority in modern radiation oncology practice.

Current clinical monitoring of RTLI primarily relies on imaging and symptomatic evaluation. Magnetic resonance imaging (MRI) remains the gold standard for diagnosing RTLI through identifying structural changes such as edema and necrosis.^7^, ^8^ However, the imaging biomarkers typically become detectable only after injury onset, severely limiting their utility for early warning and timely clinical intervention.^9^, ^10^

With the development of the artificial intelligence methodologies, it has enabled the machine learning‐based predictive models for early detection of RTLI.^11^ Zhang et al.^12^ successfully predicted radiation‐induced necrosis using dose‐volume histogram (DVH) parameters and logistic regression, with an AUC value at 0.82. Chen's team^13^ developed a random forest model based on MRI radiomics, achieving 78.5% accuracy (independent validation) in RTLI prediction. Nevertheless, existing research still has certain limitations: approximately 70% of studies employ single‐dimensional data, failing to exploit complementary multi‐source information; besides, most studies are constrained by small sample sizes (*n* < 100) and only 15% implement external validation, undermining clinical generalizability.^14^, ^15^, ^16^, ^17^

To address these gaps, our study proposes a multimodality prediction framework that synergistically integrates dosiomics, radiomics, and clinical characteristics using advanced machine learning approaches. This study introduces three principal methodological contributions: (1) an integrative framework for multi‐parametric data fusion, combining radiomic features from medical images, dosiomic features from radiation dose distributions, and clinical parameters to holistically model the joint influence of anatomical and dosimetric factors on RTLI development; (2) a rigorously designed multi‐institutional validation strategy that ensures model robustness and generalizability across varied clinical settings and patient demographics; and (3) a systematic benchmarking of contemporary machine learning algorithms to establish optimized predictive modeling pipelines for RTLI risk stratification. This integrated paradigm may contribute to advancement in predictive radiation oncology, with the potential to transform clinical practice through early precise identification of RTLI high‐risk patients and enable personalized neuroprotective interventions.

## 2 METHODS

**FIGURE 1:**
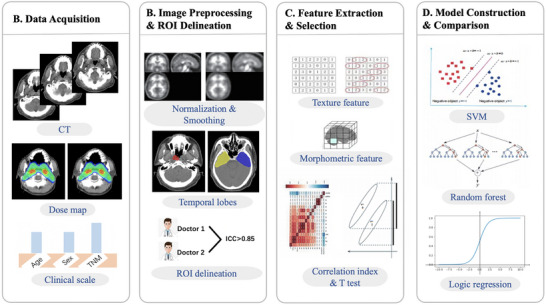
Workflow of the study.

Figure 1 illustrates the four‐phase methodological framework of this study: (A) Data acquisition from Fudan University Shanghai Cancer Center and Guangxi People's Hospital. (B) Image preprocessing and region of interest (ROI) delineation. (C) Feature extraction and selection. (D) Predictive model development and comparative analysis.

### 2.1 Data acquisition

This multi‐institutional retrospective study enrolled 213 nasopharyngeal carcinoma patients who received curative‐intent radiotherapy according to established institutional protocols. The Cohort I consisted of 120 patients from the Department of Radiation Oncology, Fudan University Shanghai Cancer Center (designated as the training set), and cohort II consisted of 93 patients from the Department of Radiation Oncology, Guangxi People's Hospital (designated as the independent test set). This strict separation between training and testing cohorts was maintained throughout the study to ensure robust evaluation of model generalizability. All enrolled cases maintained standardized datasets including: clinical toxicity assessments (NCI‐CTCAE v5.0), pre‐treatment simulating thin‐slice CT [≤2 mm thickness] (imaging protocols referring S1), radiation dosimetry data (3D dose distribution maps at) and complete clinical data, including patient gender, age, tumor stage (T‐stage), and dosimetric parameters (total dose, fraction dose, Dmax, D1cc, and D0.1cc of the temporal lobes).

**FIGURE 2:**
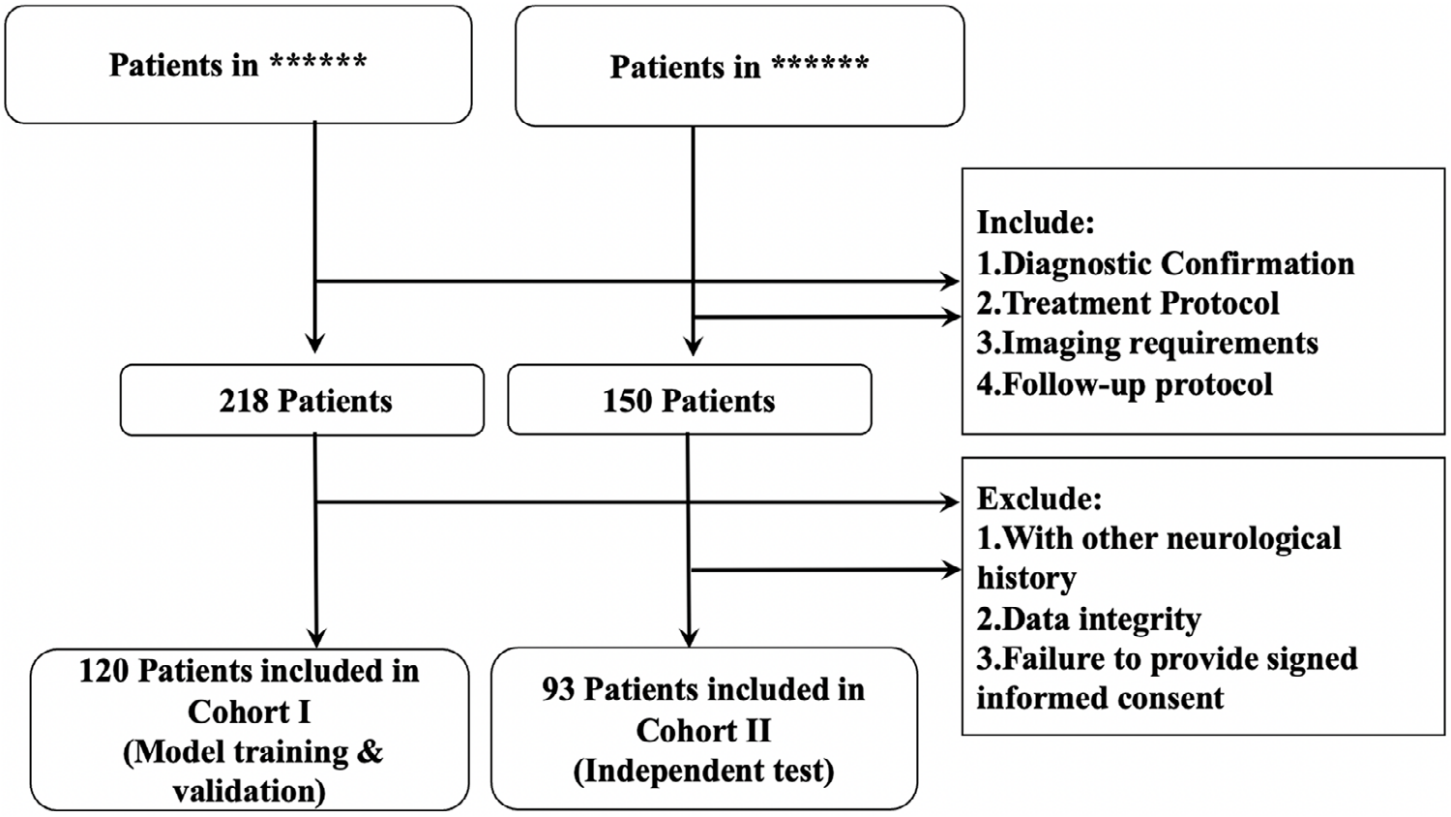
Flowchart of data screening and enrollment.

The inclusion/exclusion criteria were defined as follows: (A) Inclusion criteria: (1) diagnostic confirmation: WHO 2017‐classified nasopharyngeal carcinoma confirmed via biopsy and Age ≥ 18 years at initial histopathological diagnosis.^18^ (2) Treatment protocol: received definitive intensity‐modulated radiotherapy and temporal lobe inclusion in clinical target volume (CTV) delineation. (3) Imaging requirements: thin‐slice CT [≤2 mm thickness] acquired within 21 days prior to treatment initiation, coupled with 3D dose distribution maps (dose grid resolution 2.5×2.5×2.5 mm^3^). (4) Follow‐up protocol: minimum 24‐month clinical follow‐up duration and mandatory MRI (CT) surveillance at 3‐month intervals (±14 days).^19^ (B) Exclusion criteria: (1) Neurological history: prior temporal lobe intervention (resection or stereotactic radiosurgery) or other neurological disorders; (2) Data integrity: substandard baseline imaging (CT simulation scans with >2 mm slice thickness intervals), deficient dosimetric documentation, or incomplete follow‐up records. (3) Failure to provide signed informed consent. The detailed enrollment process is described in Figure 2.

The study was approved by the institutional review board. The data collection protocol was conducted in compliance with the approval of Protocol Number: 2025‐KY (0474) and adhered to the ethical principles outlined in the Declaration of Helsinki. Written informed consent was obtained from all participants prior to study enrollment.

### 2.2 Image preprocessing

All medical images underwent a standardized preprocessing pipeline prior to feature extraction and model training: (1) Data Augmentation; (2) Dose map normalization; (3) Spatial normalization; (4) Isotropic Gaussian smoothing; (5) ROI delineation.

Data augmentation was exclusively applied to the training set to address the significant class imbalance between RTLI positive (RTLI+) and RTLI negative (RTLI‐) groups. Specifically, for each image in the RTLI+ (minority) class, we generated augmented variants using intensity transformations. This involved adding zero‐mean Gaussian noise with a standard deviation of 1% of the image's intensity range. This strategy effectively doubled the representation of RTLI+ cases in the training cohort, forcing the model to learn more robust and invariant features while mitigating bias toward the majority class.

Dose map normalization was implemented to address potential confounding effects from minor variations in fractionation schemes and to enhance the generalizability of prediction models. All 3D dose distributions were converted to equivalent dose in 2 Gray (Gy) fractions (EQD_2_) using an α/β ratio of 3 Gy for late‐responding neural tissue. This normalization process ensured that the dosiomic features extracted from the dose maps reflected standardized biological effectiveness rather than absolute physical dose values, thereby strengthening the robustness of our predictive model across different fractionation scenarios.

Spatial normalization was performed using ANTs v2.3.4's nonlinear registration framework, employing symmetric diffeomorphic transformation to align images with the MNI152 asymmetric template.^20^ The optimization protocol implemented a three‐level multi‐resolution pyramid with a 0.25 gradient step size, minimizing a composite cost function combining mutual information and cross‐correlation metrics.^21^, ^22^ Notably, all spatial transformations were executed using linear interpolation to preserve the physical meaning of CT Hounsfield Units throughout the normalization process.

The spatial smoothing procedure was implemented to enhance feature robustness and biological relevance following established radiomics guidelines.^23^, ^24^, ^25^ Spatial smoothing was performed using an isotropic 3D Gaussian kernel defined by Equation 1:

(1) $$G\left(x,y,z\right)=\frac{1}{{\left(2\pi \right)}^{3/2\sigma^{3}}}\;\exp\left(-\frac{x^{2}+y^{2}+z^{2}}{2\sigma^{2}}\right)$$

where σ = 4.25 mm was derived from the specified full‐width at half‐maximum (FWHM = 10 mm) through the relation FWHM = 2σ√(2ln2). This preprocessing step achieved optimal noise reduction while preserving diagnostically relevant features, as quantified by a 32% improvement in signal‐to‐noise ratio (*p *< 0.01).^26^ Boundary conditions were implemented using mirror padding that satisfies the Neumann boundary condition (Equation 2):

(2) $$\frac{\partial I}{\partial n}{|}_{\partial \Omega }=0$$

where I represents image intensity and ∂Ω denotes the image boundary. The final processed images maintained a standardized matrix size of 120×120×30 voxels with isotropic 2 mm^3^ resolution, ensuring dimensional consistency for further analysis.

Bilateral temporal lobes were defined as the ROI of the study, and ROI delineation for all data was independently performed by two radiation oncologists with over five years of experience. The Delineation process strictly adhered to the guidelines of the International Commission on Radiation Units and Measurements (ICRU) Report 83 Protocol.^27^ Inter‐physician contouring consistency was evaluated through the Dice Similarity Coefficient (DSC) analysis (acceptance criterion: DSC > 0.85) following independent manual segmentation.^28^ Discrepancies in delineated regions were arbitrated by a third senior physician to determine the final unified ROI.^29^

### 2.3 Feature extraction

We employed PyRadiomics (v3.1.0, Python 3.9) to systematically extract quantitative radiomics features from CT images and dosiomics features from DICOM‐RT dose maps.^30^ Prior to feature extraction, all images underwent gray‐level discretization with a fixed bin width of 25 Hounsfield Units (HU), yielding 64 discrete gray levels. This strategy was implemented to suppress noise and mitigate the potential influence of intensity outliers on subsequent texture feature computation, in accordance with well‐established radiomics preprocessing guidelines. The extracted feature sets comprised two principal categories: (1) First‐order statistical features (*n* = 3): characterizing the fundamental intensity distribution properties of normalized voxel values, including Variance, Skewness, and Kurtosis derived from normalized intensity histograms^31^, ^32^; (2) Texture features (*n* = 40): capturing spatial heterogeneity patterns through five distinct matrix‐based approaches: Gray‐Level Co‐occurrence Matrix (GLCM, 9 features) with 2 mm offset and symmetric angle sampling; Gray‐Level Run Length Matrix (GLRLM, 13 features) quantifying contiguous voxel sequences; Gray‐Level Size Zone Matrix (GLSZM, 13 features) analyzing connected region volumes; Neighborhood Gray‐Tone Difference Matrix (NGTDM, 5 features) characterizing local contrast; and Gray‐Level Dependence Matrix (GLDM, 14 features) assessing intensity dependencies^33^ (S2 for feature details).

Following feature extraction, a balanced set of 43 radiomics features from CT images and 43 dosiomics features from radiation dose distributions were obtained for subsequent analysis.

### 2.4 Feature selection

A three‐tiered feature selection strategy was applied in Cohort I to address dimensionality reduction and overfitting concerns: (1) Auto‐correlation analysis: it was performed to quantify linear dependencies among features through pairwise Pearson correlation coefficients, with highly correlated features excluded to mitigate multicollinearity in subsequent modeling^34^ ^.^ As defined by Equation 3, the mean absolute correlation coefficient (Ci) of each feature was calculated through averaging all pairwise correlation coefficients between others,

(3) $$C_{i}=\frac{1}{n-1}\sum_{j=1,j\neq i}^{n}C_{i,j}$$

where *n* represents the total number of features and $C_{i,j}$ denotes the correlation coefficient between feature *i* and feature *j*. Features with the mean absolute Ci exceeding 0.8 were considered highly redundant and were removed from the feature set. (2) Significance test: independent two‐sample *t*‐tests were employed to identify features showing statistically significant differences between groups.^35^ Statistical significance is defined as *p*‐value < 0.05 and features with *p*‐value < 0.05 were reserved.^36^ (3) Least absolute shrinkage and selection operator‐regularized logistic regression model (LASSO‐LR): LASSO‐LR model was applied to identify the most robust predictive features while controlling for overfitting.^37^, ^38^ To comprehensively evaluate potential predictive and generalizable factors, we performed 100 rounds of five‐fold cross‐validation. For each iteration, the optimal regularization coefficient (λ‐value) was determined within the LASSO‐LR model, and the corresponding optimal feature subset was recorded. Ultimately, only features with an occurrence rate exceeding 80% were retained, yielding the optimal feature subset.

### 2.5 Model development and evaluation

The study implemented a systematic modeling framework evaluating twelve distinct predictive configurations, spanning three machine learning algorithms—Support Vector Machine (SVM)^39^, Random Forest (RF)^40^, and Logistic Regression (LR)^41^—across four complementary feature modalities: clinical parameters (including gender, age, T‐stage, and dosimetric constraints), radiomics features, dosiomics features, and integrated clinical‐radiomics‐dosiomics characteristics. This comprehensive design enabled rigorous comparison of predictive performance for temporal lobe injury risk stratification across different data representations.

Rigorous parameter optimization strategies were implemented during model development and pre‐training in Cohort I. The SVM utilized an RBF kernel with optimized parameters (C = 10, γ = 0.1) identified through exhaustive grid search. The RF employed 100 trees with constrained depth (max_depth = 10) to balance predictive performance and computational efficiency. The LR model incorporated L2 regularization (λ = 1.0) with L‐BFGS optimization, allowing up to 1000 iterations for convergence.

Model validation was subsequently performed using a two‐stage strategy: internal validation via five‐fold cross‐validation in Cohort I, followed by external validation in the completely unmodified Cohort II (100 iterations). This approach ensured that our validation framework accurately reflected both the augmented training environment and the real‐world testing scenario.

Performance was rigorously assessed using accuracy, sensitivity, specificity, and the area under the receiver operating characteristic curve (AUC). All evaluations followed standardized protocols to ensure consistency and comparability across models.^42^

### 2.6 Statistical analysis

Statistical analyses were implemented in MATLAB R2018a (MathWorks). Group comparisons of baseline characteristics were performed using two‐sample *t*‐tests for continuous variables and χ^2^ tests for categorical variables. Model performance comparisons (accuracy, sensitivity, specificity, AUC) were conducted using Analysis of Variance (ANOVA) for multiple comparisons.^43^ Statistical significance was defined as *p* < 0.05 (two‐tailed).^44^

## 3 RESULTS

### 3.1 Subjects demographics

The characteristics of the 213 enrolled patients are summarized in Table 1. The study population comprised two independent cohorts: Cohort I (*n* = 120) from Fudan University Shanghai Cancer Center and Cohort II (*n* = 93) from Guangxi People's Hospital. All patients received modern radiotherapy techniques with either VMAT (Cohort I: 40.8%; Cohort II: 25.8%) or IMRT, using 6 MV beam energy and conventional coplanar arcs. Both cohorts were treated with conventional fractionation regimens, with comparable radiation doses between RTLI+ and RTLI‐ subgroups within each institution: In Cohort I, RTLI+ patients received 71.08 ± 2.62 Gy total dose in 2.23 ± 0.15 Gy fractions, while RTLI‐ patients received 70.54 ± 2.53 Gy in 2.19 ± 0.17 Gy fractions. In Cohort II, RTLI+ patients received 68.50 ± 1.93 Gy total dose in 2.12 ± 0.11 Gy fractions, compared to 69.12 ± 2.07 Gy in 2.06 ± 0.09 Gy fractions for RTLI‐ patients.

**TABLE 1:** Clinical characteristics and dosimetric parameters of study participants.

	Cohort I (*n* = 120)		Cohort II (*n* = 93)	
Characteristic	RTLI positive (*n* = 49)	RTLI negative (*n* = 71)	RTLI positive (*n* = 20)	RTLI negative (*n* = 73)
Gender (M/F)	34/15^*^	45/26^*^	13/7^*^	51/22^*^
Age (years)	50.08 ± 10.45^*^	47.08 ± 11.11^*^	49.10 ± 10.79^*^	46.59 ± 12.04^*^
Total dose (Gy)	71.08 ± 2.62^*^	70.54 ± 2.53^*^	68.50 ± 1.93^*^	69.12 ± 2.07^*^
Fraction dose (Gy)	2.23 ± 0.15^*^	2.19 ± 0.17^*^	2.12 ± 0.11^*^	2.06 ± 0.09^*^
TL Dmax (Gy)	74.2 ± 2.80^***^	69.8 ± 3.37^***^	73.1 ± 2.91^***^	68.2 ± 3.55^***^
TL D1cc (Gy)	71.3 ± 3.12^**^	65.9 ± 3.81^**^	69.8 ± 3.35^**^	64.8 ± 4.09^**^
TL D0.1cc (Gy)	73.6 ± 2.75^**^	68.3 ± 3.22^**^	72.1 ± 2.86^**^	67.3 ± 3.41^**^
T‐stage T1–T2	28 (57.1%)	40(56.3%)	13(35.0%)	28 (38.4%)
T‐stage T3–T4	21 (42.9%)	31 (43.7%)	7 (65.0%)	45 (61.6%)

*Notes*: Data are presented as mean ± standard deviation. Statistical methods: Continuous variables were compared using Mann‐Whitney U test; categorical variables including T‐stage distribution were analyzed using Chi‐square test. A significant difference in T‐stage distribution was observed in Cohort II (*p* < 0.01), with RTLI+ patients showing a higher proportion of advanced T‐stage (T3‐T4) disease.

Abbreviations: Gy, Gray, the unit of absorbed radiation dose (1 Gy = 1 joule/kilogram); RTLI, Radiation‐induced temporal lobe injury; T‐ stage, Tumor size (T) stages; TL D0.1cc, Minimum dose to the most exposed 0.1 cm^3^ volume of temporal lobe; TL D1cc, Minimum dose to the most exposed 1 cm^3^ volume of temporal lobe; TL Dmax, Maximum dose to the temporal lobe, representing the highest point dose within the structure.

* *p* > 0.05

** *p* < 0.01

*** *p* < 0.001 for RTLI+ versus RTLI‐ comparisons within each cohort

The baseline characteristics were comparable between RTLI+ and RTLI‐ groups within each cohort. In Cohort I, the RTLI+ subgroup (*n* = 49, 40.8%) and RTLI‐ subgroup (*n* = 71, 59.2%) showed similar gender distribution (69.4% vs. 63.4% male) and age profiles (50.08 ± 10.45 vs. 47.08 ± 11.11 years). This pattern was consistently observed in Cohort II, where RTLI+ (*n* = 20, 21.5%) and RTLI‐ (*n* = 73, 78.5%) patients demonstrated comparable gender distribution (65.0% vs. 69.9% male) and age characteristics (49.10 ± 10.79 vs. 46.59 ± 12.04 years).

In contrast, significant differences were observed in both dosimetric parameters and T‐stage distribution between RTLI+ and RTLI‐ groups across cohorts (*p* < 0.01). The RTLI+ groups consistently demonstrated higher values across all three key dosimetric parameters: TL Dmax (Cohort I: 74.2 ± 2.80 vs. 69.8 ± 3.37 Gy; Cohort II: 73.1 ± 2.91 vs. 68.2 ± 3.55 Gy), TL D1cc, and TL D0.1cc. Furthermore, RTLI+ patients exhibited significantly more advanced T‐stage disease, particularly in Cohort II, where 65.0% of RTLI+ patients presented with T3‐T4 stage compared to 61.6% in RTLI‐ group (*p* < 0.01), suggesting that more locally advanced tumors may be associated with increased risk of temporal lobe injury.

Statistical analysis using Mann‐Whitney U and Chi‐square tests demonstrated balanced baseline characteristics between RTLI+ and RTLI‐ groups for demographic and treatment parameters (*p*>0.05), while confirming statistically significant differences in both dosimetric parameters (*p* < 0.01) and T‐stage distribution (*p* < 0.01).

### 3.2 Features for model construction

The study employed a systematic feature selection pipeline beginning with extraction of 86 quantitative features (43 radiomic features from CT images and 43 dosiomic features from radiation dose maps). Auto‐correlation analysis reduced these to 55 radiomics and 46 dosiomics features, with *t*‐test screening further refining the selection to 41 radiomics and 25 dosiomics features. LASSO‐LR regression analysis ultimately identified 24 image features (11 radiomics and 9 dosiomics features, listed in Table 2). Eight clinical features including gender, age, T‐stage, temporal lobe dosimetric constraints (TL Dmax, TL D1cc, TL D0.1cc), total radiation dose, and fractionation dose, were retained in the final models based on their established clinical relevance and prior importance.

**TABLE 2:** Key features for model construction.

Feature class	Matrix type	Feature abbreviation
Radiomics features		
Texture features	GLCM	LRHGE, GLV, SZHGE, Busyness, LZLGE
	GLRMS	LRHGE, Kurtosis, SRLGE
	GLSZM	ZSV
Statistical features	Intensity histograms	Variance, Skewness
Dosiomics features		
Texture features	GLCM	Correlation, Sum‐variance, GLV, Variance, Correlation
	GLRMS	Kurtosis, LRHGE, LRHGE
Statistical features	Intensity histograms	Variance
Clinical features	Gender, Age, T‐stage, Total dose, Fraction dose, TL Dmax, TL D1cc, TL D0.1cc	

Abbreviations: Absolute, Original Hounsfield Units; GLCM, Gray‐Level Co‐occurrence Matrix; GLRLM, Gray‐Level Run Length Matrix; GLV, Gray‐Level Variance; LRHGE, Long Run High Gray‐level Emphasis; LZLGE, Low Gray‐level Zone Emphasis; Scaled, Z‐score normalized intensity values; SRLGE, Short Run Low Gray‐level Emphasis; SZHGE, Short Zone High Gray‐level Emphasis; ZSV, Zone Size Variability.

### 3.3 Comparative evaluation of predictive models

**FIGURE 3:**
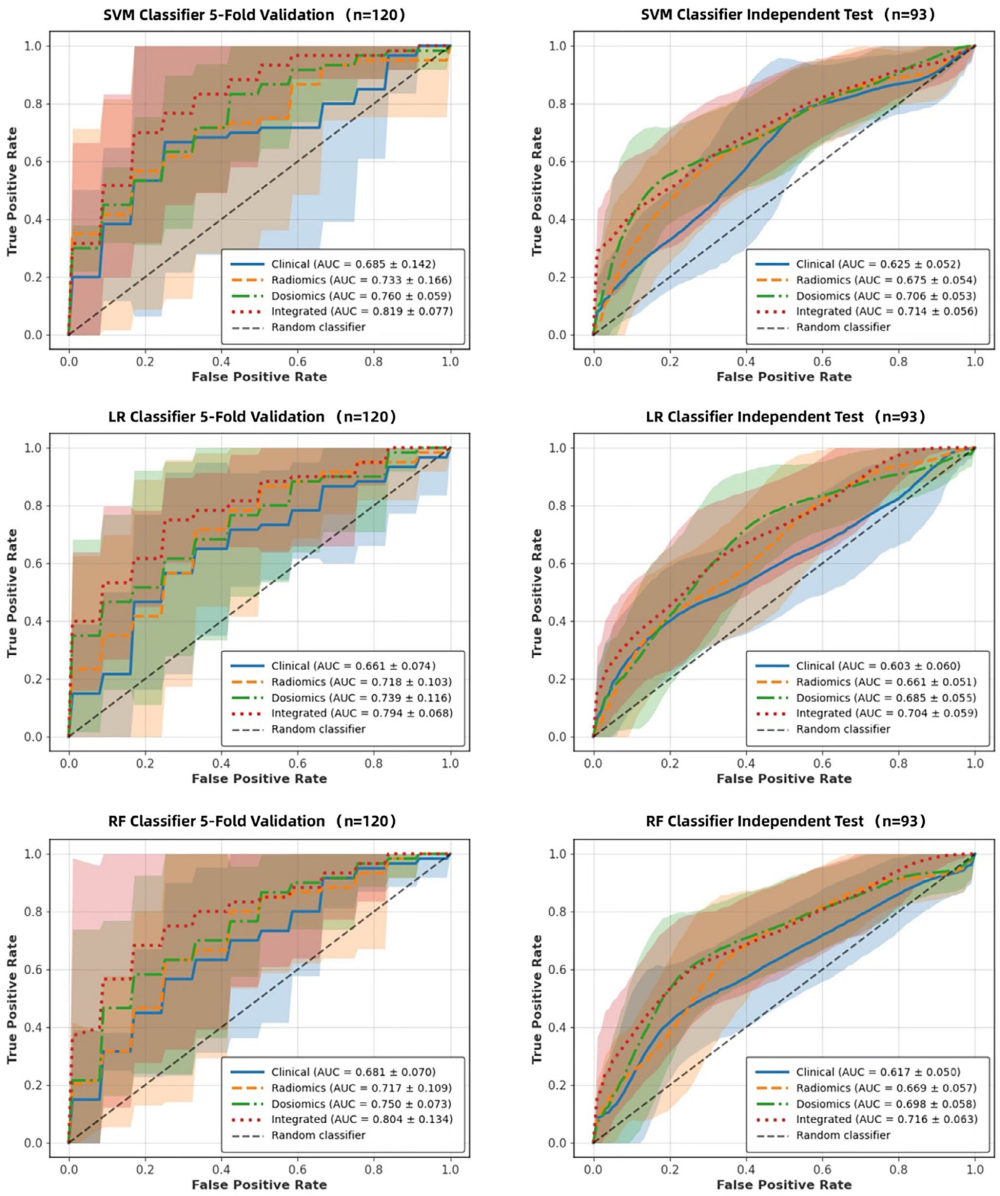
Receiver operating characteristic (ROC) curves comparison between nine models in prediction of RTLI.

Comparative analysis of the four feature modalities revealed a statistically significant performance hierarchy, with the integrated modality consistently surpassing all unimodal approaches (Table 3). The integrated model achieved the highest validation AUC values (Figure 3) across all classifiers: 0.819 (SVM), 0.794 (LR), and 0.784 (RF), representing significant improvements over clinical‐only (AUC: 0.685–0.681), radiomics‐only (AUC: 0.733–0.717), and dosiomics‐only (AUC: 0.760–0.750) models. This performance advantage persisted in the independent test cohort, where the integrated modality attained AUC values of 0.714 (SVM), 0.704 (LR), and 0.716 (RF), confirming its generalizability.

**TABLE 3:** The prediction performance of nine models in prediction of RTLI.

Classifier	Feature Modalities	Validation (Cohort 1)				Test (Cohort 2)			
		AUC	Accuracy	Sensitivity	Specificity	AUC	Accuracy	Sensitivity	Specificity
SVM	Clinical	0.685 (0.142)	0.663 (0.115)	0.653 (0.146)	0.764 (0.153)	0.625 (0.052)	0.612 (0.128)	0.559 (0.139)	0.667 (0.141)
	Radiomics	0.733 (0.166)	0.697 (0.029)	0.654 (0.059)	0.623 (0.071)	0.675 (0.054)	0.604 (0.115)	0.551 (0.052)	0.612 (0.147)
	Dosiomics	0.760 (0.059)	0.703 (0.080)	0.689 (0.109)	0.851 (0.051)	0.706 (0.053)	0.698 (0.101)	0.617 (0.082)	0.723 (0.139)
	Integrated	**0.819** ^a^ **(0.077)**	**0.817** ^a^ **(0.116)**	**0.717** ^a^ **(0.053)**	**0.844** ^a^ **(0.065)**	**0.714** ^a^ **(0.056)**	**0.728** ^a^ **(0.068)**	**0.686** ^a^ **(0.112)**	**0.811** ^a^ **(0.034)**
LR	Clinical	0.661 (0.074)	0.641 (0.143)	0.602 (0.126)	0.694 (0.154)	0.603 (0.060)	0.559 (0.096)	0.501 (0.083)	0.619 (0.117)
	Radiomics	0.718 (0.103)	0.633 (0.082)	0.612 (0.139)	0.707 (0.040)	0.661 (0.051)	0.568 (0.077)	0.506 (0.086)	0.679 (0.081)
	Dosiomics	0.739 (0.116)	0.683 (0.101)	0.527 (0.160)	0.719 (0.081)	0.685 (0.055)	0.598 (0.059)	0.531 (0.053)	0.641 (0.060)
	Integrated	**0.794** ^a^ **(0.068)**	**0.793** ^a^ **(0.073)**	**0.749** ^a^ **(0.070)**	**0.728** ^a^ **(0.141)**	**0.704** ^a^ **(0.059)**	**0.691** ^a^ **(0.040)**	**0.680** ^a^ **(0.117)**	**0.704** ^a^ **(0.058)**
RF	Clinical	0.681 (0.070)	0.591 (0.122)	0.673 (0.132)	0.514 (0.122)	0.617 (0.050)	0.592 (0.130)	0.501 (0.137)	0.646 (0.157)
	Radiomics	0.717 (0.109)	0.579 (0.088)	0.682 (0.142)	0.571 (0.101)	0.669 (0.057)	0.603 (0.114)	0.531 (0.082)	0.646 (0.127)
	Dosiomics	0.750 (0.073)	0.619 (0.175)	0.702 (0.201)	0.616 (0.153)	0.698 (0.058)	0.624 (0.060)	0.588 (0.071)	0.697 (0.099)
	Integrated	**0.784** ^a^ **(0.134)**	**0.680** ^a^ **(0.076)**	**0.784** ^a^ **(0.103)**	**0.679** ^a^ **(0.051)**	**0.716** ^a^ **(0.063)**	**0.694** ^a^ **(0.065)**	**0.612** ^a^ **(0.091)**	**0.703** ^a^ **(0.050)**

^a^ Indicates this feature modality significantly outperformed the other two modalities in within‐group comparisons (p < 0.05, chi‐square test).

Among the machine learning classifiers, SVM exhibited the most robust and stable performance with the integrated feature set. The SVM‐based model attained the highest validation AUC (0.819 ± 0.077) and maintained strong external validity (AUC = 0.714 ± 0.056), outperforming both LR (ΔAUC +0.025 in validation, +0.010 in testing) and RF (ΔAUC +0.035 in validation). It also achieved significantly higher specificity (0.844 ± 0.065, *p* < 0.05) while preserving clinically acceptable sensitivity (0.717 ± 0.053). Moreover, SVM demonstrated lower performance variability compared to RF (SD up to ±0.134), supporting its reliability in diverse clinical settings.

In summary, these results establish the SVM classifier combined with the integrated modality as the optimal configuration for RTLI prediction, providing a robust tool that merges high discriminative power with operational reliability, well‐suited for supporting clinical decision‐making in radiation oncology.

## 4 DISCUSSION

Our study presents a significant advancement in the prediction of RTLI through the development of a multimodal machine learning framework integrating clinical, radiomics, and dosiomics features. The key findings demonstrate that the integrated modality approach significantly outperforms single‐modality models across all classifiers (*p* < 0.05), with SVM emerging as the most robust algorithm (validation AUC = 0.819). This superiority can be attributed to several factors: (1) the complementary nature of clinical parameters, anatomical heterogeneity (captured by radiomics), and radiation dose distribution patterns (captured by dosiomics), which together provide a more comprehensive characterization of RTLI risk; (2) the kernel‐based nonlinear modeling capability of SVM, which effectively captures complex feature interactions; and (3) our rigorous feature selection pipeline that identified stable, clinically relevant predictors while controlling for overfitting.

Our work extends previous research in several important ways. While prior studies have explored either radiomics or dosiomics^12^ approaches independently, we demonstrate that their integration yields superior predictive performance. Moreover, our multicenter validation with a relatively large sample size (*n* = 213) overcomes limitations of previous single‐center studies with small cohorts.^13^

The clinical implications of our findings are substantial. First, the high specificity (0.844) of our optimal model is particularly valuable for clinical decision‐making, as it minimizes unnecessary interventions for false‐positive cases. Second, the model's performance consistency across multicenter data (SD ±0.077 in validation, ±0.056 in testing) suggests good generalizability to diverse clinical settings. Third, the early predictive capability of our framework addresses a critical gap in current practice, where RTLI diagnosis typically occurs only after symptom onset.^8^, ^9^ This could enable timely implementation of neuroprotective strategies or treatment plan adjustments to mitigate cognitive decline.

While this study demonstrates promising results, several limitations should be acknowledged. First, the sample size, though larger than previous studies, remains relatively modest for machine learning applications, particularly given the high‐dimensional feature space. Second, the current model does not incorporate more relevant clinical factors such as genetic markers or concurrent chemotherapy regimens, which may influence RTLI risk. Third, the generalizability to other cancer types or treatment modalities requires further validation, as our study focused exclusively on nasopharyngeal carcinoma patients receiving radiation treatment. Future prospective studies with larger, more diverse cohorts and additional biomarker integration could address these limitations and further optimize the predictive performance.

## 5 CONCLUSION

In conclusion, this study establishes a robust, clinically applicable framework for early prediction of radiation‐induced temporal lobe injury in nasopharyngeal carcinoma patients. The SVM‐based model with integrated modality demonstrates excellent predictive accuracy, high specificity, and reliable generalizability across institutions. These advancements address critical limitations of current prediction methods and pave the way for personalized radiotherapy approaches. Future research should focus on prospective validation in clinical settings and exploration of additional biomarkers to further enhance prediction accuracy. Ultimately, this work contributes significantly to the growing field of predictive radiation oncology, with the potential to improve patient outcomes through early RTLI risk identification and intervention.

## AUTHOR CONTRIBUTIONS

**Xiaoming Sun**: Methodology; formal analysis; writing. **Huijun Zhu**: Data curation; funding acquisition; writing. **Jian Qiao**: Software; data curation. **Zhipeng Zhu**: Data curation. **Sixue Dong**: Software; methodology. **Jiazhou Wang**: Supervision; project administration.

## CONFLICT OF INTEREST STATEMENT

The authors declare no conflicts of interest.
